# Non-Invasive Multimodality Imaging Directly Shows TRPM4 Inhibition Ameliorates Stroke Reperfusion Injury

**DOI:** 10.1007/s12975-018-0621-3

**Published:** 2018-03-22

**Authors:** Bo Chen, Gandi Ng, Yahui Gao, See Wee Low, Edwin Sandanaraj, Boominathan Ramasamy, Sakthivel Sekar, Kishore Bhakoo, Tuck Wah Soong, Bernd Nilius, Carol Tang, Edward G. Robins, Julian Goggi, Ping Liao

**Affiliations:** 1 Department of Research, National Neuroscience Institute, Singapore, Singapore; 2 School of Biological Sciences, Nanyang Technological University, Singapore, Singapore; 3 Singapore Institute for Clinical Sciences, Singapore, Singapore; 4 Singapore Bioimaging Consortium, Singapore, Singapore; 5 Singapore Bioimaging Consortium, Agency for Science, Technology and Research (A*STAR), 07-10 HELIOS, 11 Biopolis Way, Singapore, 138667 Singapore; 6 Department of Physiology, Yong Loo Lin School of Medicine, National University of Singapore, Singapore, Singapore; 7 Ion Channel Research Lab, Singapore, Singapore; 8 Department of Cellular and Molecular Medicine, KU Leuven, Leuven, Belgium; 9 Duke-National University of Singapore Graduate Medical School, Singapore, Singapore; 10 National Cancer Centre, Singapore, Singapore; 11 Calcium Signalling Lab, National Neuroscience Institute, 11 Jalan Tan Tock Seng, Singapore, 308433 Singapore

**Keywords:** Stroke, Reperfusion, MRI, PET, Endothelium

## Abstract

The transient receptor potential melastatin 4 (TRPM4) channel has been suggested to play a key role in the treatment of ischemic stroke. However, in vivo evaluation of TRPM4 channel, in particular by direct channel suppression, is lacking. In this study, we used multimodal imaging to assess edema formation and quantify the amount of metabolically functional brain salvaged after a rat model of stroke reperfusion. TRPM4 upregulation in endothelium emerges as early as 2 h post-stroke induction. Expression of TRPM4 channel was suppressed directly in vivo by treatment with siRNA; scrambled siRNA was used as a control. T2-weighted MRI suggests that TRPM4 inhibition successfully reduces edema by 30% and concomitantly salvages functionally active brain, measured by ^18^F-FDG-PET. These in vivo imaging results correlate well with post-mortem 2,3,5-triphenyltetrazolium chloride (TTC) staining which exhibits a 34.9% reduction in infarct volume after siRNA treatment. Furthermore, in a permanent stroke model, large areas of brain tissue displayed both edema and significant reductions in metabolic activity which was not shown in transient models with or without TRPM4 inhibition, indicating that tissue salvaged by TRPM4 inhibition during stroke reperfusion may survive. Evans Blue extravasation and hemoglobin quantification in the ipsilateral hemisphere were greatly reduced, suggesting that TRPM4 inhibition can improve BBB integrity after ischemic stroke reperfusion. Our results support the use of TRPM4 blocker for early stroke reperfusion.

**Electronic supplementary material:**

The online version of this article (10.1007/s12975-018-0621-3) contains supplementary material, which is available to authorized users.

## Introduction

Acute ischemic stroke is a major cause of morbidity and mortality worldwide. Immediate restoration of blood flow can significantly improve the chances of recovery. Currently, both fibrinolytic drug tissue plasminogen activator (tPA) and mechanical thrombectomy are used to remove the blood clot and restore cerebral circulation to the affected area [1]. The time window for successful utilization of tPA is very short after stroke onset (3 h and up to 4.5 h in certain eligible patients). Application of tPA beyond this time window leads to severe side effects, defined as ischemic reperfusion injury. Delayed reperfusion induces cerebral edema and hemorrhagic transformation, in part via disruption of the blood-brain barrier (BBB). Thus, strategies to ameliorate BBB damage are paramount. In the current study, we investigate whether inhibition of the transient receptor potential melastatin 4 (TRPM4) channel may attenuate reperfusion injury by protecting vascular endothelial cells.

TRPM4 is a voltage-dependent, non-selective monovalent cation channel which is activated by elevated cytosolic Ca^2+^ and modulated by ATP [2]. TRPM4 upregulation in vascular endothelium has previously been reported in animal models of spinal cord injury [3] and stroke [4], as well as in human stroke post-mortem brains [5]. The pathological role of TRPM4 has been well defined [3]. As a non-selective monovalent cation channel [2], TRPM4 activation leads to cell depolarization and importantly excessive Na^+^ influx causing oncotic cell death [3]. In our previous study using a permanent stroke model, TRPM4 blockade has been shown to temporarily improve motor functions [6]. However, much less is known regarding the suppression of TRPM4 expression in live animals in stroke, in particular, after ischemia reperfusion. In this study, we employed multimodality imaging techniques in a single examination to allow acquisition of co-registered complementary data to evaluate BBB integrity and subsequent reperfusion injury with a focus on stroke reperfusion in live animals.

## Materials and Methods

### Animal Model and Treatment

The creation of middle cerebral artery occlusion (MCAO) model in male Wistar rats has been described previously [6]. A detailed description can be found in the supplementary material.

### Animal Exclusion Criteria and Proper Animal Practice

Based on preliminary study, we expect to detect a 30% change in infarct reduction with a standard deviation of 5%. The animal number was calculated on the prediction of a significance of 0.05 and a power of 0.8. During the operation, nine animals died (three from siRNA-treated group, four from scrambled siRNA-treated group, and two from permanent group). For the animals that survived the operation, 8 are excluded from the study. The exclusion criteria are as follows: (1) six rats (three from siRNA group and three from scrambled siRNA group) showed no infarct formation identified by TTC staining at day 1; (2) two rats (one from siRNA group and one from scrambled siRNA group) exhibited an enhanced motor function (> 100% as compared with baseline) at day 1 post-surgery.

For better laboratory practice, treatment groups (siRNA, scrambled siRNA, and sham operation) were determined by rolling a dice and participants in the experiments were blinded to treatment. Animal operation was performed by BC and GN. Behavioral studies were carried out by SWL and YG. TTC staining and western blot was performed by SWL. Immunostaining experiments were done by GN and YG and analyzed by ES and CT. Imaging study was performed by JG, BR, and SS and analyzed by JG.

### TTC Staining, Evans Blue Extravasation, and Hemoglobin Quantification

2, 3, 5-triphenyltetrazolium chloride (TTC) staining was performed at day 1 following operation to evaluate infarct formation. After the animals were euthanized, the brains were collected, and the cerebellum and overlying membranes were removed. The brains were sectioned into 2-mm-thick coronal slices using a brain-sectioning block. The sections were stained with 0.1% TTC (Sigma, USA) solution at 37 °C for 30 min and then preserved in 4% formalin solution. The sections were scanned and the infarct size was analyzed using an image analyzer system (Scion image, Microsoft). Calculation of edema-corrected lesion was performed as described previously [7].

Blood-brain barrier permeability was assessed by measuring Evans blue extravasations 1 day after reperfusion. Evans blue (E2129; Sigma-Aldrich) of 2% concentration was injected into the jugular vein at a dose of 4 mL/kg of body weight. Six hours later, the rats were transcardially perfused with phosphate-buffered saline (PBS). Ipsilateral and contralateral hemispheres were dissected, weighted, and homogenized in 1:3 weight (mg)/volume (μl) ratios of 50% trichloroacetic acid (TCA) (T9159; Sigma-Aldrich) in saline. After centrifugation at 12,000×*g* for 20 min, supernatant was collected and thoroughly mixed with 95% ethanol (1:3) by pipetting for fluorescence spectroscopy (620 /680 nm) using the Tecan infinite plate reader. The results were quantified according to a standard curve and presented as microgram of Evans Blue per gram of tissue.

The hemoglobin volume was measured by a spectrophotometric assay. Briefly, both contralateral and ipsilateral hemispheres were dissected out after transcardial perfusion. The brain tissues were homogenized in 3 ml PBS and centrifuged at 15,000×*g* for 30 min. The supernatants were collected and incubated with Drabkin reagent (D5941; Sigma-Aldrich) for 15 min at room temperature. The optical density was quantified at 540 nm with a spectrophotometer. Hemoglobin volumes (μl) of ipsilateral and contralateral hemispheres were calculated from a standard curve obtained by adding incremental volumes of whole blood (0, 2.8, 5.6, 11.2, 22.4, 44.8 μl) to a control brain hemisphere.

### Immunofluorescent Staining and Western Blot

Immunofluorescent staining and western blot have been described in our previous publication with slight modification [8]. A detailed description can be found in the supplementary material.

### Behavioral Analysis

Motor function after MCAO was evaluated using a rotarod apparatus for rat (Ugo Basile, Italy). The performance of the rats was measured by observing the latency with which the rats fell off the rotarod. Before operation, the rats received three training trials with 15-min intervals for 5 days. The accelerating rotarod was set from 4 to 80 rpm within 10 min. The mean duration of time that the animals remained on the device was recorded 1 day before MCAO as an internal baseline control. At different time points following surgery, the mean duration of latency was recorded and compared to the internal baseline control.

### HBMECs and Scratch Assay

Human brain microvascular endothelial cells (HBMECs) was purchased from Lonza, UK. A detailed description on cell culture and treatment can be found in the supplementary material.

### Multimodality Imaging

One day after transient MCAO, cerebral injuries in live rats were evaluated by magnetic resonance imaging (MRI) and positron emission tomography (PET) scans (*n*_siRNA_ = 8, *n*_scram_ = 8, and *n*_pMCAO_ = 5). MRI imaging was performed with a 9.4 T Biospec horizontal bore magnet equipped with actively shielded magnetic field gradient coils and a linear volume coil (72 mm bore diameter; Bruker, Ettlingen, Germany). PET was performed on an Inveon PET/CT system (Siemens Inc., Washington DC). The detailed procedure can be found in the supplementary material.

### Microarray

One day after stroke reperfusion, the brains were harvested and the tissues surrounding the infarct (~ 2 mm) were collected for RNA extraction. Raw cel files were processed using standard procedures as recommended in affy packages [9]. The detailed procedure can be found in the supplementary material.

### Quantitative Real-Time PCR

Expression of claudin-1 and claudin-2 was quantified using real-time PCR as a validation of microarray results. Brain tissues surrounding the infarct area (~ 2 mm) were dissected from TRPM4 siRNA and scrambled siRNA-treated rats (MCAO 2 h, reperfusion 1 day). The detailed procedure can be found in the supplementary material.

### Statistics

All of the results are presented as the mean ± S.E.M. Student’s *t* test was used to compare two means and one-way ANOVA followed by Bonferroni’s post hoc analysis used to compare the means of data from three groups. Two-way ANOVA followed by Bonferroni post hoc test was used for Evans Blue extravasation. Repeated ANOVA with Bonferroni’s post hoc analysis was applied for behavioral studies. The results were considered significant if *p* < 0.05.

## Results

### TRPM4 Blockade in an In Vitro Model of Ischemia Reperfusion

**Fig. 1:**
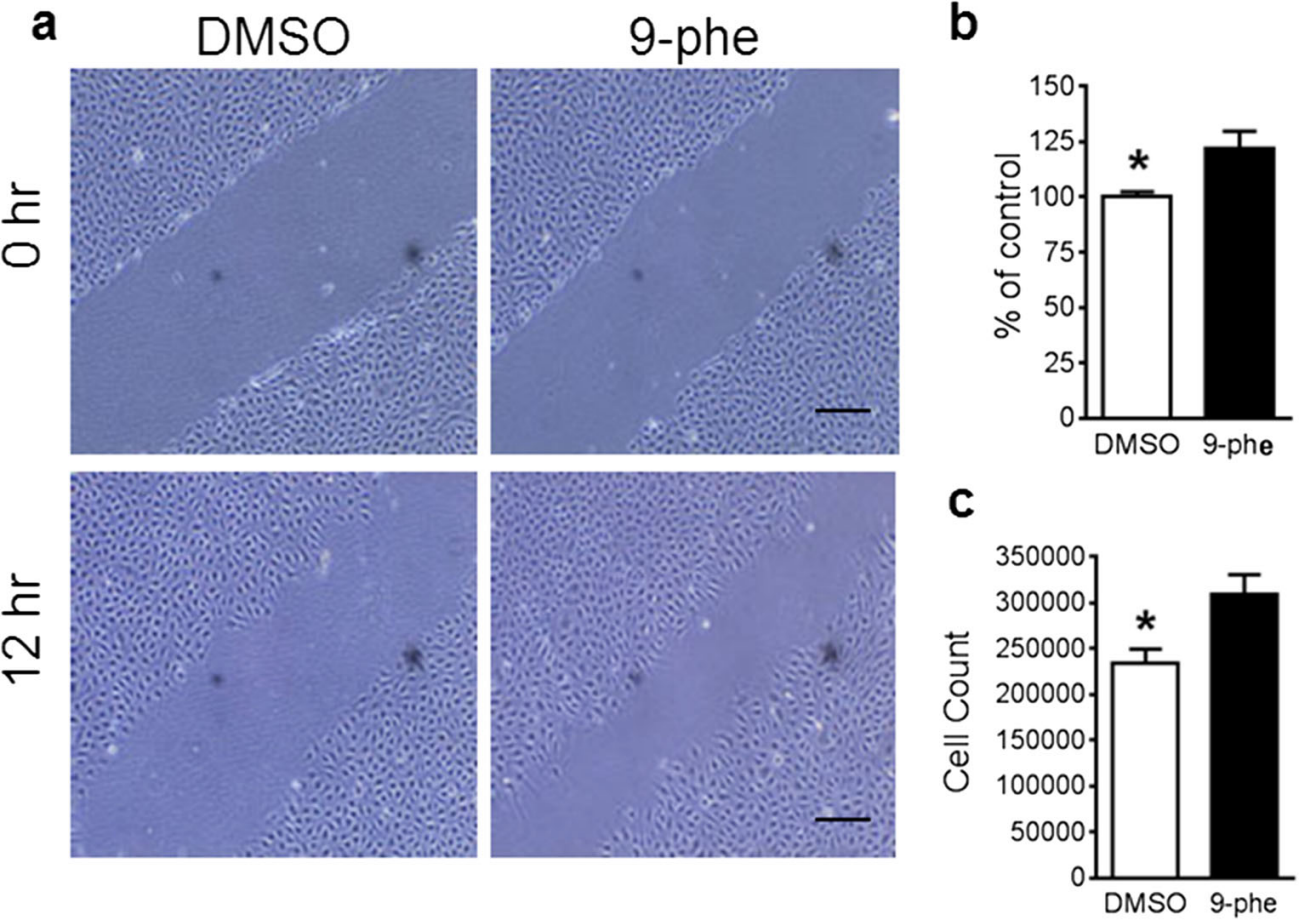
TRPM4 blockade improves human cerebral microvascular endothelial cell (HCMEC) survival and migration after hypoxia reoxygenation. **a** HCMECs were incubated in a hypoxia chamber for 5 h followed by reoxygenation and re-glucose for 12 h. Scratch wound healing assay was performed to evaluate cell growth. TRPM4 was blocked by 5 μM 9-phenanthrol (9-phe). Control cells were treated with DMSO. Scale bars 200 μm. **b** The extent of gap reduction in cells with 9-phe treatment was quantified and compared to control cells (*n* = 4, * *p* = 0.0236, Student’s *t* test). **c** Summary of cell counting (*n* = 4, *p = 0.0205, Student’s *t* test)

We first examined the effect of TRPM4 blockade on endothelial cells following ischemia reperfusion. Combined oxygen-glucose deprivation (OGD) is widely used to mimic ischemic conditions. To achieve ischemia reperfusion, human brain microvascular endothelial cells (HBMECs) near confluence were incubated under OGD condition for 5 h, followed by 12-h reoxygenation and re-glucose treatment. Prior to OGD induction, 5 mM 9-phenanthrol was added into the culture media to block TRPM4 channel [6] and equal volume of DMSO was applied into a separate petri dish as a control. Scratch assay and trypan blue exclusion test were performed to examine cell migration and survival. As shown in Fig. 1a, HBMECs migrated faster in cells with 9-phenanthrol treatment than those that received vehicle DMSO treatment. The reduction in the gap was around 20% greater in 9-phenanthrol-treated cells than in DMSO-treated cells (Fig. 1b). Further trypan blue staining showed that the cell number in the 9-phenanthrol-treated group was increased by 31.9% (Fig. 1c). The in vitro study demonstrates that TRPM4 blockade enhances vascular endothelial cell survival and growth after ischemia reperfusion.

### TRPM4 Expression in the Hyperacute Phase of Stroke

**Fig. 2:**
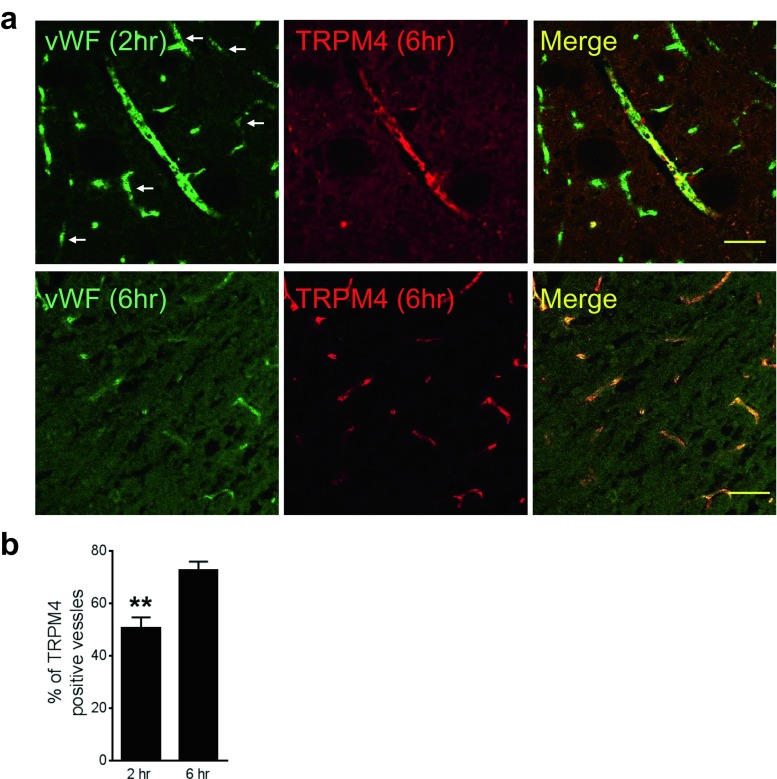
TRPM4 expression in the hyperacute phase of stroke. **a** Immunofluorescent staining of cerebral blood vessels with antibodies against vWF and TRPM4 in the ipsilateral striatum 2 h (upper panel) and 6 h (lower panel) after stroke induction. Arrows: smaller vessels do not express TRPM4 at 2 h post-stroke induction. Scale bars 50 μm. **b** Comparison of TRPM4-positive blood vessels in the ipsilateral hemispheres at 2 and 6 h post-stroke. (*n* = 6 rats, **p = 0.0109, Student’s *t* test)

**Fig. 3:**
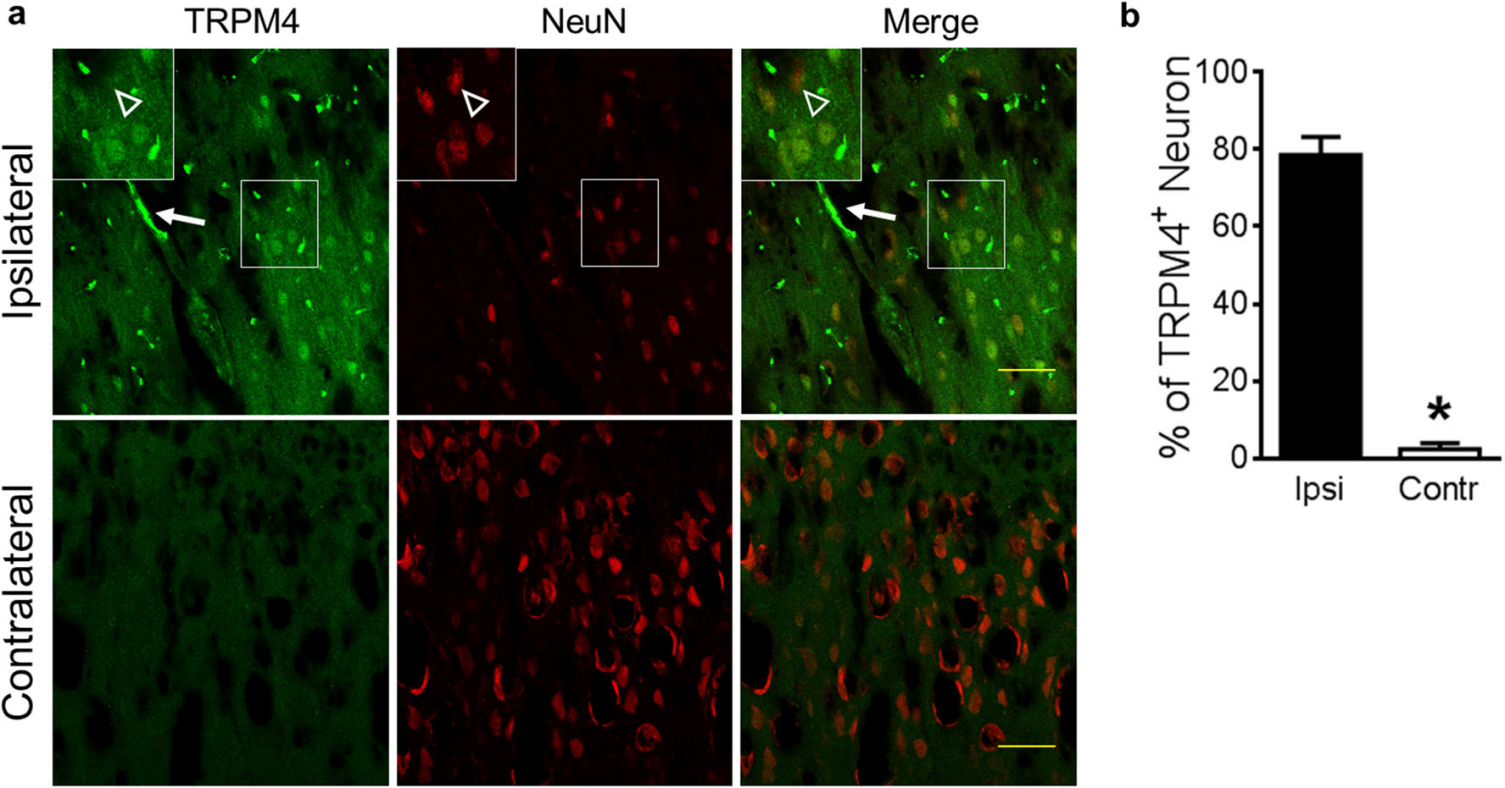
Expression of TRPM4 in neurons is heterogeneous 6 h post-stroke. **a** In contralateral cortex, TRPM4 expression is low in neurons (lower panel). In ipsilateral cortex close to the infarct core (upper panel), the expression of TRPM4 is heterogeneous. Some neurons do not express TRPM4 (arrow head). A capillary with TRPM4 expression was indicated by an arrow. Scale bars 50 μm. **b** Summary of TRPM4-positive neurons. *n* = 4, *p < 0.001, Student’s *t* test

TRPM4 is not expressed in healthy cerebral vessels [6]. When we examined the blood vessels in the hyperacute phase of stroke, it was revealed that 2 h after stroke induction, TRPM4 upregulation was clearly identified in vessels surrounding infarct core (Fig. 2a). Interestingly, TRPM4 tends to localize primarily to larger blood vessels rather than smaller ones at this early time point. In large vessels, TRPM4 distribution is also heterogeneous with certain fragment expressed a higher level of TRPM4. At 6 h post-stroke, more blood vessels including smaller ones were positively stained by anti-TRPM4 antibody (Fig. 2a). For comparison, at 2 h post-stroke, 50.6 ± 4.1% vWF-positive vessels were co-stained by anti-TRPM4 antibody which increased to 72.6 ± 3.4% by 6 h (Fig. 2b). TRPM4 upregulation was also identified in cortical neurons close to infarct 6 h post-stroke (Fig. 3a). Interestingly, the expression of TRPM4 was heterogeneous. About 80% of the neurons expressed TRPM4, which was significantly higher than the neurons in the corresponding contralateral hemisphere (Fig. 3b).

### TRPM4 Inhibition Reduced Infarction and Improved Motor Function in Transient MCAO

**Fig. 4:**
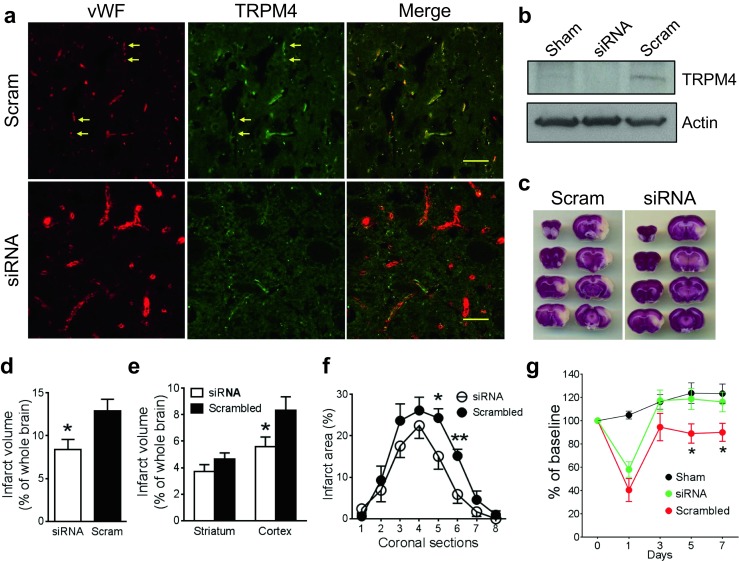
TRPM4 inhibition reduces infarction and improves motor functions. **a** Immunofluorescent staining of TRPM4 and vWF in rats receiving scrambled (Scram) or TRPM4 siRNA (siRNA) treatment. In vivo siRNA was delivered intravenously prior operation. Stroke reperfusion was achieved by 2-h MCAO and 24-h recanalization. Scale bars 50 μm. **b** Western blot was performed to detect TRPM4 expression in the ipsilateral hemispheres. Sham-operated hemisphere served as the control brain. **c** TTC staining of rat brains receiving scrambled or TRPM4 siRNA treatment. **d** Summary of the total infarct volume corrected for edema (*n*_siRNA_ = 6, *n*_scram_ = 5, *p = 0.0271, Student’s *t* test). **e** Comparison of the infarct volumes in the cortex and striatum (*n*_siRNA_ = 6, *n*_scram_ = 5, *p = 0.0476, Student’s *t* test). **f** Section-by-section infarct area distribution (*n*_siRNA_ = 6, *n*_scram_ = 5, *p = 0.0492; **p = 0.0083, Student’s *t* test). **g** Assessment of motor functions by Rotarod test (*n* = 10 per group, *p < 0.05, siRNA vs scram, repeated ANOVA with Bonferroni’s post hoc analysis)

As TRPM4 upregulation occurs at 2 h post-stroke induction, a transient stroke model was created in rats by occluding the middle cerebral artery for 2 h and reperfusion was achieved by removing the luminal thread. We have shown previously that application of 25 nmol of anti-TRPM4 siRNA successfully suppressed TRPM4 expression in a permanent MCAO model [6]. Likewise in this study, a similar dose of TRPM4 siRNA was injected intravenously prior to occlusion. Control animals received a similar dose of scrambled siRNA injection. In Fig. 4a upper panel, TRPM4 was identified in vascular endothelium in scrambled siRNA-treated animals. The vasculature tends to be of smaller size with abundant fragmentation. In TRPM4 siRNA-treated brain, TRPM4 expression was suppressed in most blood vessels (Fig. 4a lower panel) and the vessel lumen is clearly visible. Western blot further proves that in vivo TRPM4 siRNA was able to inhibit TRPM4 expression in this transient MCAO model (Fig. 4b), similar to what we observed in a permanent stroke model [6]. Cerebral infarction at 1 day after reperfusion was assessed by TTC staining. It was shown that TRPM4 siRNA significantly reduced infarct volume (Fig. 4c). The total infarct volume was reduced from 12.9 ± 1.3% in scrambled siRNA-treated animals to 8.4 ± 1.2% in TRPM4 siRNA-treated animals (Fig. 4d), representing a 34.9% reduction. Further characterization of infarct location revealed that the cortex, rather than the subcortical striatal areas, demonstrated the most infarct reduction (Fig. 4e). Section-by-section infarct area analysis showed that the major differences were located at the fifth and sixth sections, potentially associating with motor function (Fig. 4f). This result was supported by the rotarod data demonstrating that motor functions were greatly improved in TRPM4 siRNA-treated animals (Fig. 4g).

### TRPM4 Blockade Improves Cerebral Vascular Morphology

**Fig. 5:**
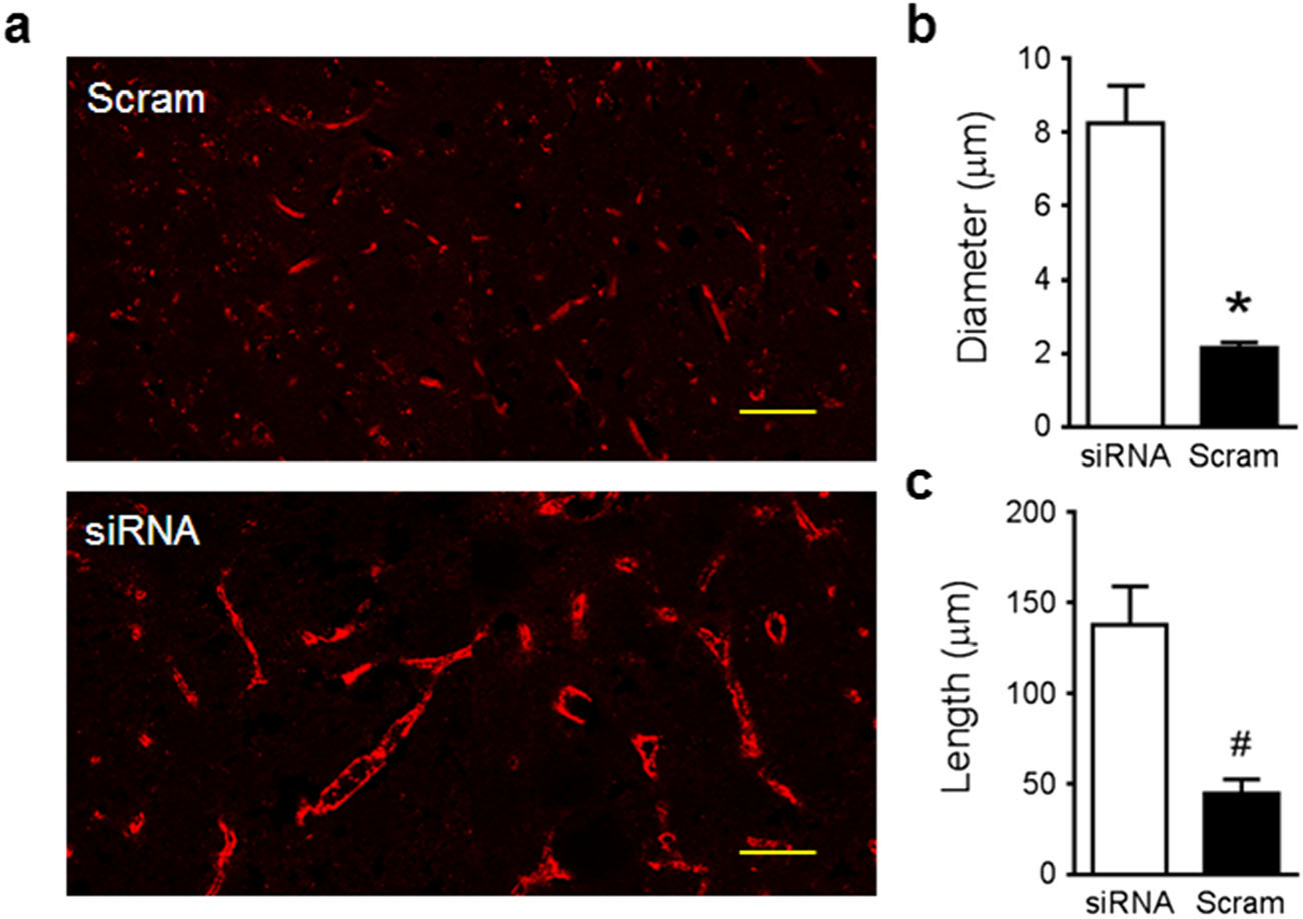
TRPM4 inhibition on vascular structure. **a** Cerebral blood vessels in the ipsilateral brains were stained by anti-vWF antibody after 2-h MCAO and reperfusion for 24 h. Scale bars 50 μm. **b**, **c** Summary of vascular diameter and length measurements in rats with scrambled or TRPM4 siRNA treatments (*n* = 14 images from 4 rats in each group, *p < 0.0001; #*p* = 0.0003, Student’s *t* test)

Vascular fragmentation, a sign of vascular damage, appeared after 1 day in a permanent stroke model and could be rescued by TRPM4 inhibition [6]. In this transient stroke model, TRPM4 suppression again greatly improved vascular morphology. In scrambled siRNA-treated animals, the size of blood vessels was generally small with prominent fragmentation. The lumen was hardly detected in most blood vessels, whereas in TRPM4 siRNA-treated animals, the lining of the capillary walls was clear and the lumen was largely intact (Fig. 5a). Next, we measured the diameter and length of the capillaries. In TRPM4 siRNA-treated animals, the diameter (8.3 ± 1 μm) was around four times of that in scrambled siRNA-treated animals (2.1 ± 0.2 μm) (Fig. 5b), and the length was around three times longer (Fig. 5c).

### TRPM4 Inhibition Protected Blood-Brain Barrier Following Stroke Reperfusion and Reduced Cerebral Edema

**Fig. 6:**
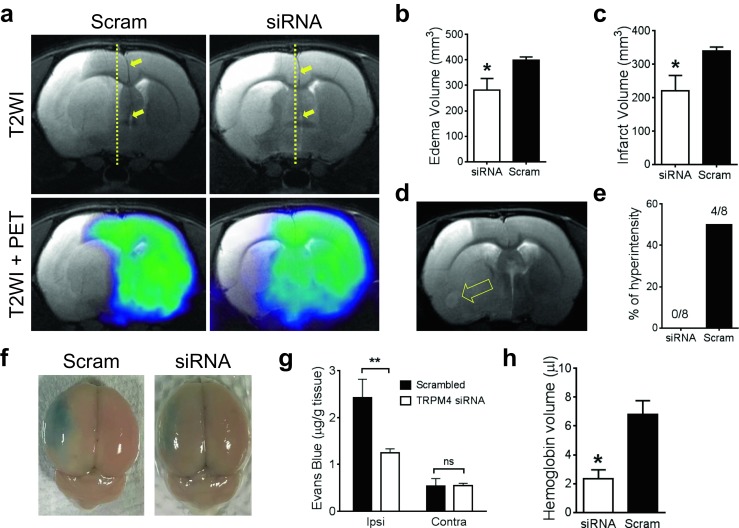
TRPM4 inhibition protects blood-brain barrier. **a** Exemplar images of T2WI and PET obtained from rats 24 h post-transient stroke induction for 2 h. Brain swelling was manifested as the midlines being shifted to the opposite hemisphere (arrows). Scrambled siRNA (scram) or TRPM4 siRNA (siRNA) were administered intravenously prior occlusion. **b** Summary of the edema volumes calculated from T2WI. *n* = 8 rats per group, *p = 0.0278, Student’s *t* test. **c** Summary of the infarct volumes identified from PET scans indicated as the brain tissues without [^18^F]FDG uptake. *n* = 8 rats per group, *p = 0.0271, Student’s *t* test. **d** An exemplar T2WI image from a scrambled siRNA-treated rat brain showing an area of hyperintensity within the infarct region (arrow). **e** Summary of rats exhibiting areas of hyperintensity. **f** Representative images of rat brains showing Evans blue extravasations. **g** Quantification of Evans blue extravasations. The results were expressed as micrograms per gram brain tissue. *n* = 3 in each group; **p < 0.01, two-way ANOVA followed by Bonferroni post hoc test. **h** Quantification of hemoglobin volume in the ipsilateral hemispheres. *n* = 6 in each group; *p = 0.0202, Student’s *t* test

As vascular injury determines stroke outcome, we examined BBB integrity using T2-weighted MRI images to quantify edema formation, a feature representing BBB disruption following ischemia reperfusion. Cerebral swelling was present in both TRPM4 siRNA and scrambled siRNA groups manifested by a shift of the midline to the opposite hemisphere (Fig. 6a upper panel). However, brain swelling was far less severe in TRPM4 siRNA-treated animals, correlating with a decrease of edema formation. Image analysis showed a significant attenuation of edema volume from 398.4 ± 13.2 mm^3^ in the scrambled siRNA-treated group to 280.7 ± 46.1 mm^3^ in the TRPM4 siRNA-treated group (Fig. 6b), representing a 30% decrease, which is similar to the reduction observed with TTC staining (Fig. 4). Interestingly, four out of eight scrambled siRNA-treated animals exhibited area of hyperintensity within the infarct core, whereas none of the TRPM4 siRNA-treated animal showed hyperintensity (Fig. 5d, e).

Together with T2-weighted imaging, positron emission tomography (PET) imaging with [^18^F]FDG was performed to measure metabolically active brain tissue following ischemia reperfusion (Fig. 6a lower panel). Total [^18^F]FDG brain tissue uptake volume was increased from 1554 ± 40.6 mm^3^ in scrambled siRNA-treated animals to 1681 ± 37.2 mm^3^ in animals with TRPM4 siRNA treatment. Brain areas with severely reduced [^18^F]FDG were considered ischemic/infarcted. The infarct volume in TRPM4 siRNA-treated animals (220 ± 46.6 mm^3^) was significantly lower than in scrambled siRNA-treated rats (338.8 ± 12.3 mm^3^) (Fig. 6c), representing a 34.9% reduction. This result again correlates well with the 34% reduction as determined by TTC staining (Fig. 4).

To further characterize BBB disruption, we assessed Evans blue extravasation 24 h after reperfusion. In contralateral hemispheres, very small amount of Evans blue was detected. However, in ipsilateral hemisphere, Evans blue extravasation, corrected for the baseline reading, was significantly attenuated by TRPM4 siRNA treatment (Fig. 6f, g). As hemorrhagic transformation is an important feature of BBB disruption following reperfusion, we quantified hemoglobin volume in the ipsilateral hemisphere. Again, TRPM4 siRNA treatment significantly reduced hemorrhage at 1 day following stroke reperfusion (Fig. 6h).

**Fig. 7:**
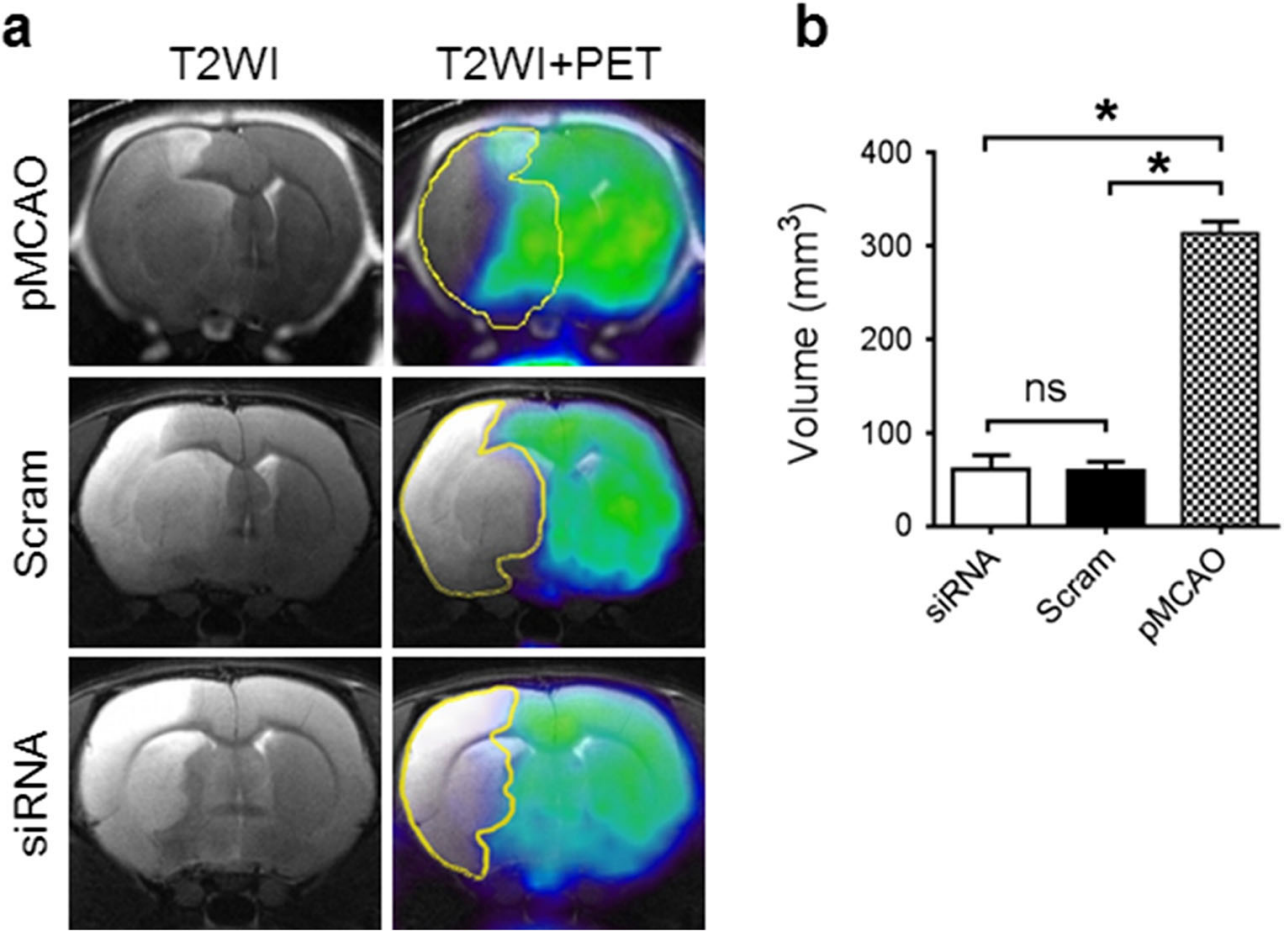
Comparison of penumbral regions identified by MRI and PET. **a** Exemplar MRI and PET images from permanent and reperfusion stroke models. Images were taken at 1 day after operation. The occlusion time for reperfusion model is 2 h. Edema areas were labeled in yellow lines. The animals in the permanent MCAO group received no treatments. The results showed that [^18^F]FDG uptake was evidenced in substantial edematous tissues in rats with permanent MCAO. **b** Comparison of the penumbral areas which were identified as the edematous tissue with [^18^F]FDG uptake. *p < 0.0001, ns no significance, *n* = 8 in each group, one-way ANOVA followed by Bonferroni post hoc test

Next, MRI and PET data from permanent and transient stroke model animals were co-registered and assessed for differences in penumbral tissue volume which is defined as the edematous tissue with [^18^F]FDG uptake. In the permanent MCAO model, large areas of brain tissue displayed both edema formation and metabolic activity (Fig. 7a). However, in the transient MCAO model, both the scrambled and TRPM4 siRNA-treated animals exhibited much smaller penumbral regions. The penumbral area in the permanent stroke model is significantly higher than those in the transient models (Fig. 7b). No significant difference was observed between the TRPM4 siRNA- and scrambled siRNA-treated groups. These in vivo data collectively demonstrate that TRPM4 inhibition could protect BBB integrity and reduce tissue damage following ischemia reperfusion.

### TRPM4 Inhibition Enhanced Claudin-1 and Claudin-2 Expression

**Fig. 8:**
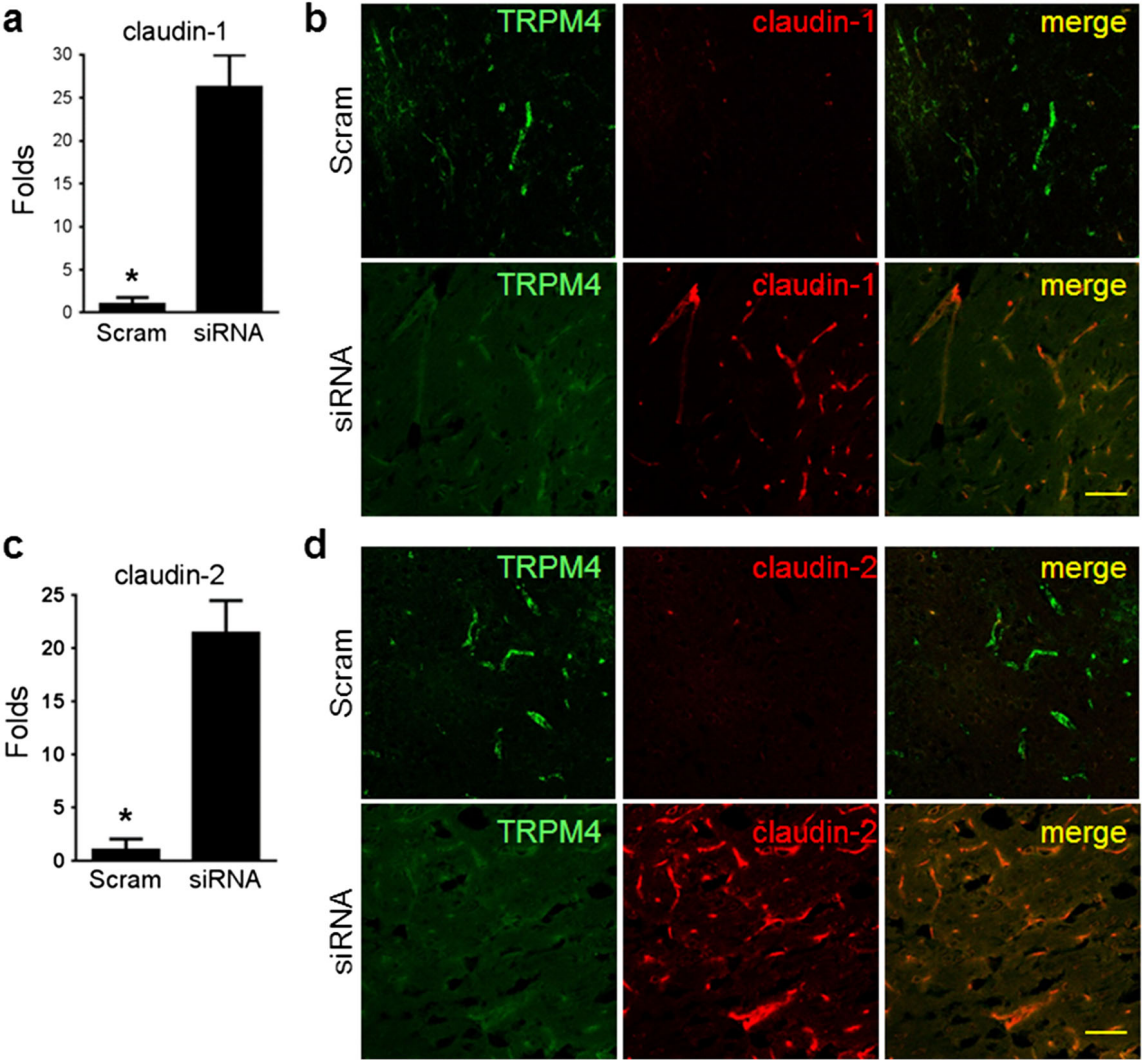
TRPM4 inhibition induces de novo expression of tight junction molecules claudin-1 and claudin-2 in endothelial cells. **a** Real-time PCR quantification of claudin-1 in brain tissues surrounding the infarction. Expression of the transcripts was normalized to β actin before calculation of the relative expression level using the comparative Cq-method (∆∆Cq). Scram: scrambled siRNA. siRNA: TRPM4 siRNA. *n* = 3, *p = 0.0021, Student’s *t* test. **b** Double staining of claudin-1 and TRPM4 in capillaries close to the infarction. **c** Real-time PCR quantification of claudin-2. *n* = 3, *p = 0.0027, Student’s *t* test. **d** Double staining of claudin-2 and TRPM4 in capillaries close to the infarction. Scale bars 50 μm

To further investigate how TRPM4 inhibition affects BBB integrity following stroke, we examined the expression of tight junction (TJ)-related proteins in brain tissues surrounding the infarct using microarray. Data analysis revealed that claudin-1 and claudin-2 were upregulated upon TRPM4 suppression. Real-time PCR further confirmed that the transcripts of claudin-1 and claudin-2 were drastically upregulated in TRPM4 siRNA-treated animals (Fig. 8). Immunofluorescent staining was thus carried out to examine the expression of claudin-1 and claudin-2 proteins. For better comparison, the samples were processed together under similar conditions, including the same antibody dilutions and applying the same exposure time for confocal microscopy. In scrambled siRNA-treated animals, the expression of claudin-1 and claudin-2 was hardly detected, whereas both proteins were upregulated in capillaries in the TRPM4 siRNA-treated animals. Interestingly, the expression of claudin-1 and claudin-2 is inversely correlated with TRPM4 protein, suggesting that TRPM4 inhibition could enhance BBB integrity by upregulating novel tight junction proteins.

### TRPM4 Blockade Accelerated Vascular Recovery

**Fig. 9:**
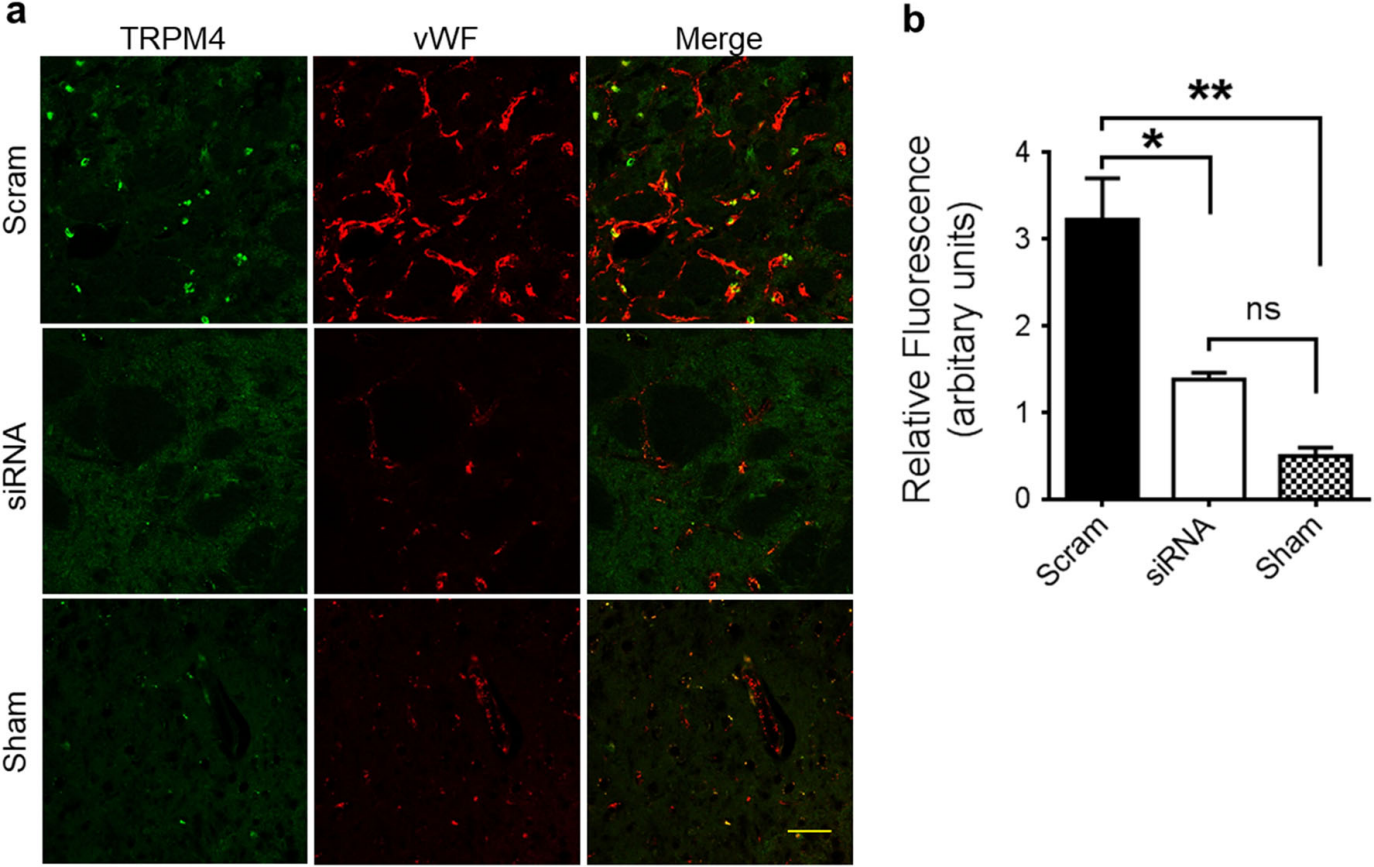
TRPM4 suppression accelerates vascular recovery. **a** Fourteen days following stroke reperfusion, vasculatures within the infarct areas were co-stained with anti-TRPM4 and anti-vWF antibodies and compared with healthy tissue from sham-operated animals. Scram scrambled siRNA. siRNA TRPM4 siRNA. Sham sham operation. Scale bar 50 μm. **b** Quantification of vWF fluorescence intensity. *n* = 3, *p < 0.05; **p < 0.01, ns no significance. One-way ANOVA followed by Bonferroni post hoc test

To examine the effect of TRPM4 inhibition at a later time point, rat brains at 14 days were harvested and examined. In sham-operated brain (Fig. 9a), vascular TRPM4 was not detected, similar to our previous report [6]. In scrambled siRNA-treated brain, capillaries in the infarct area were strongly stained by anti-vWF antibody with TRPM4 sparsely stained. This transient expression of TRPM4 correlates with our previous study [6]. Conversely, the staining pattern of cerebral vessels in TRPM4 siRNA-treated animals (Fig. 9a) was similar to sham-operated animals. No TRPM4 staining was identified in the vessels. Interestingly, the staining of vWF was strong in scrambled siRNA-treated sample as compared to sham-operated or siRNA-treated samples (Fig. 9a, b). Quantification of vWF fluorescence showed no difference between sham-operated and siRNA-treated samples (Fig. 9b). Upregulated vWF expression is an indicator of hypoxia-induced endothelial stimulation [10]. Therefore, we can conclude that vascular recovery is accelerated by TRPM4 inhibition.

## Discussion

Reperfusion therapy was first used in managing ischemic stroke patient within 3 h of stroke onset [11]. After years of examination of its efficacy and safety, the time window has been extended to 4.5 h in certain eligible patients [12]. Even though the global outcome was improved with thrombolysis, the incidence of intracranial hemorrhage caused by vascular injury was still higher in patients with thrombolysis than with placebo [11, 12]. Therefore, the need for a novel way of vascular protection, even in patients with early stroke onset, remains urgent. In the present study, we identified that TRPM4 channel was upregulated as early as 2 h post-stroke induction. The differential expression of TRPM4 in endothelium and neurons at early stage of stroke suggests that the effect of hypoxia is heterogeneous among various cells and locations.

Using a 2-h MCAO reperfusion model, we evaluate the effect of TRPM4 suppression on edema formation and tissue infarction in live animals for the first time using multimodality imaging technique. A reduction in edema formation was identified in rats receiving TRPM4 siRNA, accompanied by an increase of metabolically active tissue compared to scrambled siRNA-treated animals. Attenuated edema is indicative of improved BBB integrity during ischemia reperfusion. The reduction in tissue damage, measured by TTC staining, T2-weighted MRI, and [^18^F]FDG-PET imaging was very consistent (around 30%), strongly suggesting that TRPM4 inhibition in this transient stroke model could salvage significant amounts of brain tissue. The protection of BBB integrity by TRPM4 inhibition was further supported by the results showing that none of the TRPM4 siRNA-treated animals exhibited areas of hyperintensity within the infarct core, a sign of severe BBB leakage which occurred in 50% of control animals. Imaging of the tissues surrounding the infarct core revealed interesting differences between the permanent and transient models. In permanent stroke, [^18^F]FDG was taken up by substantial amounts of edematous tissue surrounding the infarct core. Surprisingly, in the transient stroke model, only small areas of tissue surrounding the infarct core showed the signs of both edema and [^18^F]FDG uptake. These results indicate that the brain tissues salvaged by TRPM4 inhibition after stroke reperfusion are well protected, likely due to the blood resupply.

In addition to imaging live animals, multiple lines of evidence from both in vitro and in vivo experiments support the use of TRPM4 blockers in vascular protection. In cultured HBMECs, TRPM4 inhibition enhanced endothelial survival and migration which is in line with previous study showing that TRPM4 blockade prevented lipopolysaccharide-induced endothelial cell death [13]. Furthermore, a reduction of Evans blue extravasation and hemoglobin quantification was observed within the ipsilateral hemispheres, suggesting that TRPM4 inhibition could alleviate reperfusion-associated BBB disruption. Morphological examination of the vasculature revealed more exciting results. In our previous study using a permanent stroke model, although the vessels with TRPM4 siRNA treatment were longer and smooth, the diameter of the capillaries was similar to that observed in control animals [6] with no visible lumen, indicating a lack of functional blood flow in these vessels, whereas in the current transient stroke model, the capillary diameter in TRPM4 siRNA-treated animals was much larger and displayed clear lumen in most blood vessels. Indeed, the diameter of capillaries after TRPM4 siRNA treatment (8.3 ± 1 μm) is more representative of normal perfused capillaries (~ 4–8 μm) [14], suggesting that these blood vessels could conduct functional blood flow. Interestingly, TRPM4 inhibition could also accelerate vascular recovery. vWF, an indicator of hypoxia-induced endothelial stimulation [10], was found downregulated by TRPM4 siRNA treatment at 14 days post-operation. Furthermore, as TRPM4 is found upregulated in neurons and astrocytes [15], TRPM4 inhibition could contribute to the survival of these cells. We identified an upregulation of TRPM4 in cortical neurons close to the infarct, but not in the contralateral hemisphere. Interestingly, the TRPM4 expression pattern was heterogeneous. About 80% of the neurons express TRPM4, suggesting that hypoxic impact varies among neurons close to the infarct core. Therefore, blocking TRPM4 channel may help improve the survival of these neurons.

This study identified for the first time a novel mechanism of TRPM4 suppression in improving BBB integrity. We observed that TRPM4 inhibition could enhance the expression of TJ components claudin-1 and claudin-2. In control animals, the expression of claudin-1 and claudin-2 is extremely low which is consistent with previous studies showing that claudin-3, claudin-5, and claudin-12, rather than claudin-1 and claudin-2, are major components of TJ in BBB [14]. In brain, claudin-1 and claudin-2 are primarily expressed in epithelial cells, including choroid plexus. Claudin-2 has not been found in endothelial TJ and claudin-1 expression in the TJ is controversial [14]. It is unlikely that the induction of claudin-1 and claudin-2 is due to hypoxia as their expression was low in scrambled siRNA-treated animals, and previous studies failed to show a link between hypoxia and the induction of claudin-1 expression in HBMEC [16]. Therefore, our data indicate that the expression of claudin-1 and claudin-2 may be a result from TRPM4 suppression after stroke. De novo expression of claudin-1 and claudin-2 in vascular endothelium is beneficial to BBB integrity. It has been reported that in contrast to occludin, claudin-1 or claudin-2 were able to reconstitute de novo TJ strands in TJ-free mouse fibroblasts [17]. Furthermore, ectopic expression of claudin-1 has been shown to seal BBB TJs in experimental autoimmune encephalomyelitis [18]. Thus, in ischemia reperfusion, induction of claudin-1 and claudin-2 may strengthen BBB integrity by forming additional TJs, which could be a novel therapeutic mechanism of TRPM4 inhibition. Further experiments are needed to verify the exact underlying mechanisms.

To suppress TRPM4 expression in the hyperacute stage of stroke, TRPM4 siRNA was delivered at the start of the operation in this study. Ideally, TRPM4 blockers should be administered before recanalization. TRPM4 blocker 9-phenanthrol is a metabolized product of highly toxic polycyclic aromatic hydrocarbon (PAH) phenanthrene and thus is unlikely to be used in clinical practice as it has the potential to be concentrated in tissues [19]. Currently, sulfonylureas such as glibenclamide have been shown to be promising TRPM4 blockers. TRPM4 has been reported to form a channel complex with sulfonylurea receptor-1 (SUR1), an auxiliary subunit of K_ATP_ channel [15], and SUR1 blocker sulfonylureas have been used to treat brain diseases such as stroke [20]. However, there is controversy regarding the regulatory role of SUR1 on TRPM4 and the effect of sulfonylureas in stroke treatment [21–24]. A recent review has summarized the preclinical findings on glibenclamide for the treatment of stroke in various stroke models [25]. In clinical practice, SUR1 blocker sulfonylureas are widely used to manage diabetes mellitus. Multiple studies on stroke patients with or without diabetes mellitus revealed that use of sulfonylureas before or after stroke onset could reduce hemorrhage transformation, attenuate cerebral edema, and improve neurological outcome [20, 22]. In contrast, some studies on diabetic patients with stroke showed that application of sulfonylureas achieved no better outcome than other anti-diabetic treatments [26, 27]. Such controversies may arise from differences in patient inclusion criteria, dose of sulfonylureas, or the severity of diabetes mellitus.

Interestingly, a recent study showed that application of sulfonylurea glimepiride achieved neuroprotection against stroke only in normal mice but not in type 2 diabetic mice [28], suggesting that the presence of diabetes mellitus could be a confounding factor for the use of sulfonylureas to manage stroke. To avoid the effect of sulfonylureas on diabetes mellitus, antagonists that act directly on TRPM4 channel is another good option.

Ischemia reperfusion injury is a major confounding factor for reperfusion therapy in stroke. Delayed reperfusion induces cerebral edema and hemorrhagic transformation, in part via disruption of the BBB. This study provides robust evidence in support of using TRPM4 blockers to ameliorate reperfusion injury. Mitigating edema formation will surely improve stroke outcome in patients receiving reperfusion therapy. Next critical experiment is to examine whether TRPM4 inhibition could extend current reperfusion time window, which is limited by the severe reperfusion injury during delayed recanalization. As TRPM4 inhibition can protect vasculature and improve BBB integrity following stroke reperfusion, TRPM4 blockers may potentially extend current time window for reperfusion therapy when applied in together with tPA or other recanalization treatments, thereby offering great therapeutic management for stroke patients who arrive at hospitals late.

## Conclusions

The current study indicates that TRPM4 inhibition improves BBB integrity and ameliorates reperfusion injury. TRPM4 is an ideal target for stroke treatment as it is not expressed in brain tissue; thus, blockade of TRPM4 is unlikely to affect normal brain functions. Furthermore, possibilities of severe side effects on other tissues or organs are also low. Therefore, injection of TRPM4 blockers before recanalization may constitute a new strategy to mitigate reperfusion injury.

## Electronic Supplementary Material

ESM 1 (DOCX 28 kb)

## References

[1] Lo EH, Ning M (2016). Mechanisms and challenges in translational stroke research. J Investig Med.

[2] Vennekens R, Nilius B (2007). Insights into TRPM4 function, regulation and physiological role. Handb Exp Pharmacol.

[3] Gerzanich V, Woo SK, Vennekens R, Tsymbalyuk O, Ivanova S, Ivanov A, Geng Z, Chen Z, Nilius B, Flockerzi V, Freichel M, Simard JM (2009). De novo expression of Trpm4 initiates secondary hemorrhage in spinal cord injury. Nat Med.

[4] Zhang E, Liao P (2015). Brain transient receptor potential channels and stroke. J Neurosci Res.

[5] Mehta RI, Tosun C, Ivanova S, Tsymbalyuk N, Famakin BM, Kwon MS, Castellani RJ, Gerzanich V, Simard JM (2015). Sur1-Trpm4 cation channel expression in human cerebral infarcts. J Neuropathol Exp Neurol.

[6] Loh KP, Ng G, Yu CY, Fhu CK, Yu D, Vennekens R, Nilius B, Soong TW, Liao P (2014). TRPM4 inhibition promotes angiogenesis after ischemic stroke. Pflugers Arch.

[7] Walberer M, Stolz E, Muller C, Friedrich C, Rottger C (2006). Experimental stroke: ischaemic lesion volume and oedema formation differ among rat strains (a comparison between Wistar and Sprague-Dawley rats using MRI). Lab Anim.

[8] Liao P, Yu D, Hu Z, Liang MC, Wang JJ, Yu CY, Ng G, Yong TF, Soon JL, Chua YL, Soong TW (2015). Alternative splicing generates a novel truncated Cav1.2 channel in neonatal rat heart. J Biol Chem.

[9] Gautier L, Cope L, Bolstad BM, Irizarry RA (2004). Affy—analysis of affymetrix gene chip data at the probe level. Bioinformatics.

[10] Aird WC (2007). Phenotypic heterogeneity of the endothelium: I. Structure, function, and mechanisms. Circ Res.

[11] The National Institute of Neurological Disorders and Stroke rt-PA Stroke Study Group (1995). Tissue plasminogen activator for acute ischemic stroke. N Engl J Med.

[12] Hacke W, Kaste M, Bluhmki E, Brozman M, Davalos A (2008). Thrombolysis with alteplase 3 to 4.5 hours after acute ischemic stroke. N Engl J Med.

[13] Becerra A, Echeverria C, Varela D, Sarmiento D, Armisen R (2011). Transient receptor potential melastatin 4 inhibition prevents lipopolysaccharide-induced endothelial cell death. Cardiovasc Res.

[14] Zlokovic BV (2008). The blood-brain barrier in health and chronic neurodegenerative disorders. Neuron.

[15] Simard JM, Woo SK, Schwartzbauer GT, Gerzanich V (2012). Sulfonylurea receptor 1 in central nervous system injury: a focused review. J Cereb Blood Flow Metab.

[16] Mark KS, Davis TP (2002). Cerebral microvascular changes in permeability and tight junctions induced by hypoxia-reoxygenation. Am J Physiol Heart Circ Physiol.

[17] Furuse M, Sasaki H, Fujimoto K, Tsukita S (1998). A single gene product, claudin-1 or -2, reconstitutes tight junction strands and recruits occludin in fibroblasts. J Cell Biol.

[18] Pfeiffer F, Schafer J, Lyck R, Makrides V, Brunner S (2011). Claudin-1 induced sealing of blood-brain barrier tight junctions ameliorates chronic experimental autoimmune encephalomyelitis. Acta Neuropathol.

[19] Koenig S, Porte C, Sole M, Sturve J (2013). Biliary PAH and alkylphenol metabolites, biomarker enzyme activities, and gene expression levels in the deep-sea fish Alepocephalus rostratus. Environ Sci Technol.

[20] Sheth KN, Simard JM, Elm J, Kronenberg G, Kunte H (2016). Human data supporting glyburide in ischemic stroke. Acta Neurochir Suppl.

[21] Favilla CG, Mullen MT, Ali M, Higgins P, Kasner SE (2011). Sulfonylurea use before stroke does not influence outcome. Stroke.

[22] Kunte H, Busch MA, Trostdorf K, Vollnberg B, Harms L, Mehta RI, Castellani RJ, Mandava P, Kent TA, Simard JM (2012). Hemorrhagic transformation of ischemic stroke in diabetics on sulfonylureas. Ann Neurol.

[23] Sala-Rabanal M, Wang S, Nichols CG (2012). On potential interactions between non-selective cation channel TRPM4 and sulfonylurea receptor SUR1. J Biol Chem.

[24] Woo SK, Kwon MS, Ivanov A, Gerzanich V, Simard JM (2013). The sulfonylurea receptor 1 (Sur1)-transient receptor potential melastatin 4 (Trpm4) channel. J Biol Chem.

[25] Caffes N, Kurland DB, Gerzanich V, Simard JM (2015). Glibenclamide for the treatment of ischemic and hemorrhagic stroke. Int J Mol Sci.

[26] Horsdal HT, Mehnert F, Rungby J, Johnsen SP (2012). Type of preadmission antidiabetic treatment and outcome among patients with ischemic stroke: a nationwide follow-up study. J Stroke Cerebrovasc Dis.

[27] Weih M, Amberger N, Wegener S, Dirnagl U, Reuter T, Einhäupl K (2001). Sulfonylurea drugs do not influence initial stroke severity and in-hospital outcome in stroke patients with diabetes. Stroke.

[28] Darsalia V, Ortsater H, Olverling A, Darlof E, Wolbert P, Nystrom T, Klein T, Sjoholm A, Patrone C (2013). The DPP-4 inhibitor linagliptin counteracts stroke in the normal and diabetic mouse brain: a comparison with glimepiride. Diabetes.

