# Supplementary material for: Non-Invasive Multimodality Imaging Directly Shows TRPM4 Inhibition Ameliorates Stroke Reperfusion Injury
Source: Transl Stroke Res. 2018 Mar 22;10(1):91–103. doi: 10.1007/s12975-018-0621-3 (PMC6327008; doi:10.1007/s12975-018-0621-3)
Supplement: Supplementary file 1 — (DOCX 28 kb) [file 12975_2018_621_MOESM1_ESM.docx]

**SUPPLEMENTAL MATERIAL**

Non-invasive multimodality imaging directly shows TRPM4 inhibition ameliorates stroke reperfusion injury

Bo Chen*, MD, PhD; Gandi Ng*, BSc; Yahui Gao, BSc; See Wee Low, BSc; Edwin Sandanaraj, MSc; Boominathan Ramasamy, PhD; Sakthivel Sekar, PhD; Kishore Bhakoo, PhD; Tuck Wah Soong, PhD; Bernd Nilius, MD, PhD; Carol Tang, PhD; Edward G Robins, PhD; Julian Goggi, PhD; Ping Liao, MD, PhD.

From Department of Research, National Neuroscience Institute, Singapore (B.C., G.N., Y.G., S.W.L., E.S., C.T., P.L.); School of Biological Sciences, Nanyang Technological University, Singapore (E.S.); Singapore Institute for Clinical Sciences (E.S.) and Singapore Bioimaging Consortium, Agency for Science, Technology and Research (A*STAR) (B.R., S.S., K.B., E.G.R., J.G.); Duke-National University of Singapore Graduate Medical School (C.T., P.L.); National Cancer Centre, Singapore (C.T.); Department of Physiology, Yong Loo Lin School of Medicine, National University of Singapore (T.W.S.); and Ion Channel Research Lab, Department of Cellular and Molecular Medicine, KU Leuven, Belgium (B.N.).

* These authors contribute equally in this study.

**SUPPLEMENTAL METHODS**

**Transient stroke model**

The protocol was approved and performed in accordance with the guidelines of the Institutional Animal Care and Use Committee of the National Neuroscience Institute, Singapore. Prior to the surgery, the male Wistar rats (250 - 300 g) were anesthetized with ketamine (75 mg/kg) and xylazine (10 mg/kg) intraperitoneally. The left common carotid artery (CCA) was exposed and temporarily ligated using a vascular clip (Aesculap, B. Braun, Germany). The left external carotid artery (ECA) and internal carotid artery (ICA) were then dissected from the surrounding tissues. Occipital artery and superior thyroid artery (branches of ECA) were occluded, following with ECA ligation. The extracranial branch of ICA was temporarily blocked with a vascular clip. The distal end of the ECA was cut and a silicon coated filament (0.37 mm, Cat #403756PK10, Doccol Corp, Redlands, CA) was introduced into the ICA through the ECA stump. Subsequently, the suture around the ECA stump was tightened, and the microvascular clip was removed. The filament was then gently advanced from the ECA to the ICA lumen for approximately 18-20 mm. The ligation on the CCA was released lastly after the suture around the ECA-intraluminal filament was tightened. Relative regional cerebral blood flow of the animals were monitored by Laser-Doppler flowmetry (moorVMS-LDF2™, Moor Instruments Inc., DE, USA). Animals with ≥80% flow reduction were included in the study. To achieve reperfusion, the filament was removed from the ICA 2 hours after occlusion, followed by the closure of ECA. Successful recanalization was verified by Laser-Doppler flowmetry. The sham-operated rats underwent similar procedures, except for the insertion of the suture. TRPM4 *in vivo* Ready siRNA and scrambled siRNA were purchased from Ambion, Life Technologies Corporation, USA. Prior to the occlusion, 25 nmole siRNA was delivered intravenously.

**Immunofluorescent staining and western blot**

Rats were sacrificed and perfused with normal saline and subsequently 4% paraformaldehyde. The brains were collected and sectioned at 10 µm of thickness. After washing with 0.2% Triton X-100 phosphate-buffered saline (PBST), 100 µl of blocking serum (10% goat serum and 1% bovine serum albumin in 0.2% PBST) was added onto the slides for 1 h. The sections were then incubated with primary antibodies overnight at 4 °C. The primary antibodies used in the study are: anti-TRPM4 (sc-27540, Santa Cruz and TA500381, Origene), anti-von Willebrand factor (vWF) (AB7356, Millipore and AB6994, Abcam). On the following day, tissue sections were washed 3 times with TNT wash buffer (0.1 M Tris-HCl buffer, pH 7.5, containing 0.15 M NaCl and 0.05% Tween 20). The slides were then incubated with FITC-conjugated or Texas red-labelled secondary antibodies for 1 h at room temperature. After washing three times with wash buffer, the slides were mounted with FluorSave™ reagent (Merck). The results were visualized using laser scanning confocal microscope system (Fluoview BX61, Olympus). The negative control was processed in an identical procedure except for primary antibody incubation, and no positive signal was identified.

Brain tissues from the ipsilateral hemispheres were harvested for western blot. Protein concentration was determined by Pierce BCA Protein Assay Kit (23227; Life Technologies). 80 µg of total protein was resolved on 12% SDS-PAGE gels at 80V, and electrophoretically transferred to Immun-Blot PVDF membranes (1620177; Bio-Rad) at 110V for 2h at 4°C. After blocking with StartingBlock (PBS) blocking buffer (37538; Life Technologies) for 1 h at room temperature, membranes were incubated overnight at 4°C with a 1:300 dilution of primary antibody against TRPM4 (ACC-044; Alomone) and a 1:5000 dilution of primary antibody against actin (A1978; Sigma-Aldrich). The membranes were then washed and incubated with a 1:5000 dilution of the respective secondary antibody (A4416; A4914; Sigma-Aldrich) for 1 h at room temperature. Primary and secondary antibodies were both prepared in StartingBlock (PBS) blocking buffer with 0.05% Tween^®^20 (P7949; Sigma-Aldrich). Protein bands were detected using the Amersham ECL Western Blotting Analysis System (RPN2109, GE Healthcare) and visualized using a medical x-ray processor (MXP-2000; KODAK). Quantification was done using ImageJ.

***In vivo* siRNA delivery**

TRPM4 in vivo Ready siRNA and scrambled siRNA (Cat #: 4404020) were purchased from Ambion, Life Technologies Corporation, USA. The sequences of TRPM4 siRNA were as follows: sense 5’-CGCUAGUAGCAGCAAAUCUtt-3’ and antisense 5’-AGAUUUGCUGCUACUAGCGtg-3’. siRNA was dissolved in water, and 25 nmole siRNA was injected intravenously into the rat prior to the occlusion.

**Human Brain Microvascular Endothelial Cells (HBMECs) and scratch assay**

HBMECs (Lonza, Wokingham, UK) were cultured at cell density of 1x 10^4^ cells/well in 6-well culture plates (Corning, USA) coated with Attachment Factor (CSC Certified, 4Z0-210) at 37°C with 5% CO_2_. Both experiment and control plates from the same batch of cells were cultured concurrently under a similar condition. The culture medium is endothelial growth medium-2 (EGM-2) consisting of endothelial basal medium (EBM), supplemented with 2% fetal bovine serum, hydrocortisone, hFGF, VEGF, R3-IGF-1, ascorbic acid, HEGF, GA-1000, and heparin. When cells reached confluence, 4 straight scratches were made using a p10 pipet tip in each quarter of the culture plate and photomicrograph was taken at time 0 under a phase-contrast light microscope for each scratch. Detached cells and debris were washed away three times with PBS. To achieve oxygen/glucose deprivation (OGD), EGM-2 was changed to EBM without fetal bovine serum or growth supplements. To block TRPM4 channel, 9-phenanthrol from a stock solution 0.2 M dissolved in dimethyl sulfoxide (DMSO) was added into the wells to reach a final concentration of 5 µM. Equal volume of DMSO was added into the control plate. Hypoxia was induced by incubating the cells in a hypoxic chamber (Stem Cell Technologies, Vancouver, Canada) with 1% O_2_ and 5% CO_2_ at 37°C. 5 hours later, the cells were transferred back into a normal incubator and the media was changed to EGM-2 without 9-phenanthrol treatment. After reoxygenation for 12 hours, photomicrographs were taken. For cell counting, a separate set of experiment was performed under the same condition. The cells were harvested and counted using the trypan blue exclusion method. The number of cells was quantified by a light microscopy using a haemocytometer chamber. To quantify cell migration, the width of each scratch was measured and normalized to the same scratch before OGD. A reduction in the gap in 9-phenanthrol treated group was calculated and compared to the respective control group. A number larger than 100 suggests a more reduction in the gap than the control group. This experiment was repeated 4 times.

**MRI Imaging**

Magnetic resonance imaging was performed on all animals to assess cerebral edema post stroke. MRI imaging was performed with a horizontal bore magnet, operating at 400 MHz (7 T) with a 22 cm diameter (Bruker Cliniscan, Ettlingen, Germany), and equipped with actively shielded magnetic field gradient coils and a linear volume coil (72 mm bore diameter; Bruker). T2-weighted MRI was acquired without contrast enhancement. Rapid imaging (three images in axial, coronal and sagittal orientations; FLASH sequence; repetition time TR/TE = 10/2 ms; nominal resolution 0.18×0.18×1.02 mm^3^, acquisition time = 9 s) was performed for subsequent slice positioning. The entire brain volume was acquired using a fast T2w sequence (RARE sequence with rare factor of 9; TR/TE_eff_ = 2500/58 ms; number of experiments = 4 with 12 contiguous slices; nominal resolution 0.12×0.12×0.70 mm^3^; acquisition time = 12 min). A three-dimensional (3D) FLASH sequence was acquired with the same transaxial and rostrocaudal field of view (FOV) as the microPET FOV, i.e. 100×100×127 mm (TR/TE_eff_ = 400/4.81 ms; NEX = 1; nominal resolution 0.78×0.78×0.78 mm^3^; acquisition time = 1 min) for co-registration. Total acquisition time per animal was approx. 20 min. MRI imaging dates were coregistered with the PET imaging for all experiments. Hyperintense regions on the T2 weighted images were outlined to evaluate the spatial development of the oedema volume in the rat brains. Oedema volume was calculated from the T2 weighted images by drawing a region of interest around the infarct border manually on each slice and multiplying by the slice thickness (Syngo Fast View, Siemens USA).

### Small-Animal PET/CT Imaging

Small-animal PET imaging was performed on an Inveon PET/CT system (Siemens Inc., Washington DC). Images were generated from sinogram data, rebinned to 2-dimensional format by the Fourier rebinning algorithm, followed by 2-dimensional filtered back projection. The microPET scanner was calibrated in terms of absolute activity concentration (kBq/cm^3^) by imaging a phantom approximating the dimensions of a rat body and filled with a known concentration of [^18^F]fluoride.

For the ‘static’ imaging protocols, the [^18^F]FDG animals were fasted for 8 hours and images were acquired at 50-70 min post injection (p.i.). Approximately 20 MBq of the ^18^F-labelled radiopharmaceuticals were injected via the lateral tail vein (in an injection volume of not more than 5 mL/kg). The animals were anaesthetised using inhalational isoflurane at 2% alveolar concentration and fixed using stereotactic ear bars to a custom made PET imaging bed (ASI instruments, Warren, MI) for acquisition of the PET data and a CT scan was performed for anatomical placement and attenuation correction (40 kV, 500 µA; 4x4 binning, ~100 µm resolution, ensuring a total radiation dose within safe exposure limits of ~15mGy, thus limiting radiation effects as much as possible). Images were reconstructed using the image reconstruction, visualization, and analysis software supplied by the manufacturer. Small animal PET and CT data were analyzed by using Amide software (Sourceforge 10.1, http://amide.sourceforge.net). The PET, CT and MRI images were co-registered to confirm anatomical location of uptake. Uptake of radioactivity in the brain was determined by placement of a Volume of Interest (VOI) delineated using the MRI images and presented as percent injected dose/gram (%ID/g).

**Microarray**

Raw cel files were processed using standard procedures as recommended in affy packages [^1^](#_ENREF_1). The signal intensities of core probe sets of GeneChips were normalized using RMA (robust multichip averaging) algorithm. The processed values were log2 transformed and evaluated for data distributions across the samples. A linear model was fitted to study the differential pattern across our experimental conditions ^[2](#_ENREF_2)^. A log2 fold change of 1.25 was applied to discern transcripts in TRPM4 siRNA treated rats. The transcripts were annotated using Rat gene transcript cluster database from R/Bioconductor packages [^3^](#_ENREF_3). The core transcripts mapped with entrez identifiers were further considered for pathway enrichment analysis. MetacoreTM from Thomson Reuters (New York, NY) pathway database was interrogated with TPRM4 signature to identify top enriched pathways. We applied gene ontology process filters including cell activation, cell adhesion, cell ageing and cell chemotaxis in order to find the characteristic relevance of our signature in cellular pathways background. The enriched p-values in pathway analysis were further adjusted for multiple corrections in hypergeometric test. A false discovery rate (FDR)–adjusted P value of less than 0.05 was defined as statistically significant in microarray-based analysis.

**Quantitative real-time PCR**

Expression of claudin-1 and claudin-2 was quantified using real-time PCR as a validation of microarray results. Brain tissues surrounding the infarct area ( ̴2 mm) were dissected from TRPM4 siRNA and scrambled siRNA treated rats (MCAO 2hours, Reperfusion 1day). Total RNA was isolated using RNeasy Mini Kit (Qiagen, USA). The concentration the RNA was determined using a Nanodrop (Thermofisher). 18-mer oligodT was used to synthesize cDNA with SuperScript® II Reverse Transcriptase (Invitrogen, Thermofisher). The primers for claudin-1, claudin-2 and β-actin were listed below. cDNA samples were amplified using FastStart Essential DNA Green Master (Roche applied science) on a LightCycler® 96 System (Roche applied science). Duplicate experiments were carried out for validation. Amplification and melting conditions were as follows: 10 min at 95 °C, 40 cycles of 95 °C for 15 s, 60 °C for 30s, 72 °C for 30s, and melting at a gradient from 65°C to 97°C at 0.02°C per second, fluorescence acquisitions per 1°C. Data was obtained from the LightCycler® 96 software. The specificity of real-time PCR was confirmed by analysing the melting-curves. Relative expressions were determined by normalizing claudin-1 and claudin-2 Ct values to the control β-actin Ct value. Data were finally analysed by the 2^-∆∆Ct^ method [^4^](#_ENREF_4).

Primers used for amplifying Claudin-1, Claudin-2, and β-actin.

|  | Forward primer (5′–3′) | Reverse primer (5′–3′) |
| --- | --- | --- |
| Rat claudin-1 | AGTACTTTGCAGGCAACCAG | TGGTGGACACAAAGATTGCG |
| Rat claudin-2 | AAAACTTCAGCAGCGGCAAG | AAGCATGGGGATGAAATGGC |
| Rat β-actin | GCAGGAGTACGATGAGTCCG | ACGCAGCTCAGTAACAGTCC |

**References**

1. Gautier L, Cope L, Bolstad BM, Irizarry RA. Affy--analysis of affymetrix genechip data at the probe level. *Bioinformatics*. 2004;20:307-315

2. Ritchie ME, Phipson B, Wu D, Hu Y, Law CW, Shi W, Smyth GK. Limma powers differential expression analyses for rna-sequencing and microarray studies. *Nucleic acids research*. 2015;43:e47

3. Durinck S, Moreau Y, Kasprzyk A, Davis S, De Moor B, Brazma A, Huber W. Biomart and bioconductor: A powerful link between biological databases and microarray data analysis. *Bioinformatics*. 2005;21:3439-3440

4. Livak KJ, Schmittgen TD. Analysis of relative gene expression data using real-time quantitative pcr and the 2(-delta delta c(t)) method. *Methods*. 2001;25:402-408
